# The Influence of Sex, Ear, and Age on Auditory Brainstem Responses Recorded with the NeuroAudio System

**DOI:** 10.3390/diagnostics16070971

**Published:** 2026-03-24

**Authors:** Milaine Dominici Sanfins, Maria Eduarda Aidar Santillo, Mariana Ferreira Pires Martins, Diego Lourenço dos Santos Silva, Elzbieta Gos, Piotr Henryk Skarzynski, James W. Hall

**Affiliations:** 1 Department of Speech-Hearing-Language, Universidade Federal de São Paulo, São Paulo 04044-020, SP, Brazil; 2 Department of Teleaudiology and Screening, World Hearing Center, Institute of Physiology and Pathology of Hearing, Kajetany, 05-830 Warsaw, Poland; 3 Department of Otolaryngology, Institute of Sensory Organs, 05-830 Warsaw, Poland; 4 Osborne Audiology, Salus at Drexel University, Elkins Park, PA 19027, USA

**Keywords:** auditory brainstem response, sex, ear, references values, electrophysiology

## Abstract

**Background:** Clinical interpretation of the auditory brainstem response (ABR) relies on precise normative data. While our previous work provided evidence of sex-based differences in ABR latencies in a normative sample (*N* = 73), this larger-scale investigation (*N* = 244) validates these findings and extends them to other features of the ABR, such as wave amplitude and interaural latency asymmetry. **Methods:** This retrospective, cross-sectional study collected ABRs from 244 participants aged 3–79 years (134 men, 110 women) with normal hearing. All underwent basic audiological assessment and click-evoked ABR measurement with the NeuroAudio system. Wave latencies, interpeak intervals, and amplitudes were analyzed. **Results:** Absolute latencies for wave III and wave V, and all interpeak latency intervals, were significantly shorter in women versus men (*p* ≤ 0.001). No statistically significant sex-based differences were found for wave I and V amplitudes. Statistically significant right versus left ear differences were found for wave V absolute latency and for interpeak intervals I–III and I–V, with the left ear consistently showing prolonged responses compared to the right. No significant interaural differences were identified for waves I and III, or for the III–V interval. **Conclusions:** This study confirms the significant effect of sex on ABR temporal parameters, but not on wave amplitudes. There were also significant interaural asymmetries. These findings support the use of sex-specific, and potentially ear-specific, normative data to maximize diagnostic accuracy in audiology.

## 1. Introduction

The ABR is a valuable clinical procedure for auditory and neurological diagnosis as it provides objective information about the integrity of the auditory pathways from the 8th cranial nerve through to the brainstem. Valid ABR data can be obtained from patients who are sleeping naturally, sedated, or under general anesthesia. As a result, the ABR is an essential audiological tool for the auditory assessment of difficult-to-test populations, such as infants, young children, or individuals of any age with cognitive or communication difficulties [1,2].

Research and clinical findings over the past five decades have led to evidence-based test protocols and normative data [3,4,5,6]. However, additional normative studies are needed because of technological advancements and the introduction of new instrumentation on the market. One new device is the NeuroAudio evoked response system (Neurosoft, Ivanovo, Russia) that is available in Europe, Latin America, and Asia. Sanfins et al. (2022) [3] reported the first normative study with the NeuroAudio device. Click-evoked ABR data were collected from 73 individuals over 3 years of age. The results confirmed sex-based differences in ABR latencies, differences which had been reported since the 1970s [7,8,9]. In comparison to men, women systematically exhibited shorter latencies, a finding that has been replicated across multiple population samples and research contexts [10]. The underlying cause of these sex-related differences remains a topic of active investigation. Possible factors include structural differences in the auditory system, neurobiological differences, and hormonal differences [7,8,11]. The presence of sex differences is one factor that must be considered when clinically interpreting ABR data [8,12].

Using a substantially larger clinical sample and the NeuroAudio device, the present study was designed to expand and confirm previous investigations examining the effect of sex on ABRs. In this latest work, we also looked at the amplitudes of waves I and V, the ratio of the amplitudes of waves V/I, and the interaural latency differences in wave V.

## 2. Material and Methods

### 2.1. Ethics Statement

The present study used a retrospective and cross-sectional design, and was approved (protocol number 7.510.718) by the Research Ethics Committee of the Federal University of São Paulo (UNIFESP). All participants provided an Informed Consent Form (ICF) in writing. Data collection was conducted at the facilities of the Electrophysiology Laboratory of the Department of Speech Therapy at the Paulista School of Medicine, Federal University of São Paulo (UNIFESP/EPM).

### 2.2. Participants

ABR data were analyzed from 244 individuals aged 3 to 79 years (134 men and 110 women). Participants met the following requirements: (1) age over 3 years; (2) normal bilateral otoscopy; (3) normal hearing sensitivity (pure tone hearing thresholds < 20 dB HL from 250 to 8000 Hz [13]; (4) normal speech audiometry results, defined as a Speech Recognition Threshold (SRT) consistent with pure tone average and word recognition scores within normal limits (88–100%) at 40 dB above the pure tone average for 0.5, 1, and 2 kHz in each ear; (5) type A tympanograms (Jerger, 1970) characterized by peak compliance of 0.3–1.3 mmhos over the range −100 to +200 daPa [14]; and (6) ipsilateral and contralateral acoustic reflexes in both ears detected with a probe tone of 226 Hz in response to pure tones of 0.5, 1, 2, and 4 kHz) [14].

### 2.3. Procedures

All participants underwent pure tone and speech audiometry. Audiological assessment was conducted by an experienced audiologist. Air conduction pure tone audiometry was conducted for octave frequencies from 0.25 to 8 kHz, plus 3 and 6 kHz. Bone conduction pure tone audiometry was performed at 0.5, 1, 2, and 4 kHz. Normal hearing sensitivity for air- and bone-conducted pure tones was defined by thresholds ≤ 20 dB HL (WHO, 2021) [13]. Speech audiometry included estimation of speech recognition threshold (SRT) with a list of two-syllable words. SRT was defined as the intensity at which the participant correctly repeated 50% of the presented words. Word recognition was measured with monosyllable words presented at 40 dB above the pure tone average for 0.5, 1, and 2 kHz. All participants had normal word recognition scores (88–100%) for each ear.

Audiological evaluations were conducted in an acoustic sound booth using a Pello^TM^ audiometer (GSI, Eden Prairie, MN, USA) and TDH 39 headphones, calibrated according to ISO-389 and IEC-645. Acoustic immittance measurements, as well as ipsilateral and contralateral acoustic reflexes, were evaluated at 226 Hz using a TympStar Pro 2^TM^ middle ear analyzer (GSI, Eden Prairie, MN, USA) [14].

Data collection took place in an electrically shielded and acoustically attenuated environment while the participant rested comfortably in a reclined position. The Neuro-Audio system (Neurosoft, Ivanovo, Russia) was used for ABR measurement. ABRs were recorded with a three-electrode setup with the non-inverting electrode on the high forehead (Fz), the inverting electrode on the ipsilateral mastoid, and the ground electrode on the forehead [15]. Electrode impedance was <5 kΩ, and the inter-electrode impedance was maintained below 3 kΩ. Participants were instructed to close their eyes throughout the procedure to minimize artifacts from eye movements. Stimuli consisted of a 0.1 ms rarefaction click presented monaurally via ER-3A insert earphones (Etymotic Research, Inc., Schaumburg, IL, USA). The presentation level was 80 dB nHL at a repetition rate of 19.3/s. Ear testing order was randomized across participants. Two separate collections of 2000 artifact-free stimuli were averaged per ear. Responses were band-pass filtered from 0.1 to 3 kHz.

Evaluations were conducted by an audiologist who had experience in auditory electrophysiology exceeding 25 years. Researchers visually identified and marked the principal waveform peaks (I, III, and V), and from them were derived the interpeak latencies I–III, III–V, and I–V, the interaural differences in wave V latencies, and the ratios of the V/I amplitudes.

## 3. Results

### 3.1. Statistical Analysis

Statistical analysis was carried out using IBM Statistics SPSS version 24. The assumption of normality was checked with a Kolmogorov–Smirnov test. The assumption was not met, so nonparametric tests were applied: *χ*^2^ test, Mann–Whitney *U*-test, Wilcoxon test, and Spearman rank correlation. Statistical significance was specified as *p*-values less than 0.05.

### 3.2. Effect of Sex

There were 244 patients comprising 134 men and 110 women aged 3 to 79 years. The distribution of sexes was similar (*p* = 0.124) but the age difference between them was statistically significant (*U* = 5541.0; *p* = 0.001).

Table 1 displays ABR latencies for men and women. With the exception of wave I, there were sex differences in both absolute latencies and inter-wave latency intervals for all ABR waves from the right and from the left ear.

### 3.3. Effect of Age

**Figure 1:**
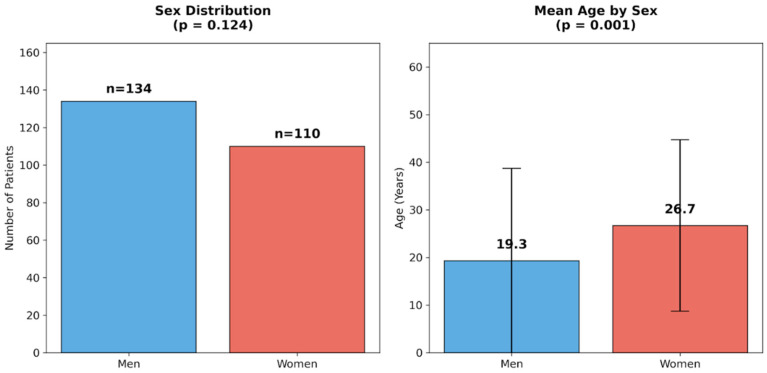
The distribution of sexes and mean age by sex.

There were 244 patients, comprising 134 men and 110 women. The distribution of sexes was similar (*p* = 0.124). The ages of men ranged from 3 to 79 years (*M* = 19.3, *SD* = 19.4) while the ages of women ranged from 3 to 72 years (*M* = 26.7, *SD* = 18.0). The age difference between sexes was statistically significant (*U* = 5541.0; *p* = 0.001) see Figure 1.

The individuals were divided into 3 age groups: 3–17 years (men = 88, women = 40), 18–49 years (men = 30, women = 60), and over 50 years (men = 16, women = 10). Table 2 shows a statistically significant positive correlation with age for wave V latency in both ears (right: *rho* = 0.21, left: *rho* = 0.33), indicating an age-related prolongation of wave I. The correlations between age and intervals involving wave I are a secondary consequence of the age-related prolongation of wave I latency. For the I–III interpeak interval, age showed a significant negative correlation in both ears (right: *rho* = −0.31; left: *rho* = −0.30). The negative direction reflects the fact that wave I latency increases with age, whereas wave III latency remains relatively stable. As a result, the difference between the two latencies decreases, producing a shorter I–III interval in older individuals. The same was true for the I–V interval in the right (*rho* = −0.31) and the left (*rho* = −0.32) ears.

There was no statistically significant correlation between wave III latency and age. But there was a significant correlation between age and wave V latency. It was weakly negative and reached statistical significance only in the left ear (*rho* = −0.14, *p* < 0.05), while the association in the right ear was negligible and non-significant (*rho* = −0.09). Statistically, this reflects a small inverse relationship between age and wave V latency.

Given the wide age range in the study sample (3–79 years), the latter correlation was examined in greater detail. As before, the sample was divided into three age groups: 3–17 years (*n* = 128), 18–49 years (*n* = 90), and 50 years and above (*n* = 26). Within these age groups, the correlations between age and wave V latency were re-evaluated (Table 3).

In the youngest age group (3–17 years), age showed a significant negative correlation with wave V latency in both ears (right: *rho* = −0.30, *p* < 0.01; left: *rho* = −0.34, *p* < 0.001). This indicates that within this developmental period, increasing age is associated with shorter wave V latencies.

In the adult group (18–49 years), the direction of the association reversed. Age showed a significant positive correlation with wave V latency (right: *rho* = 0.29, *p* < 0.01; left: *rho* = 0.28, *p* < 0.01), meaning that in this age group, older individuals had longer wave V latencies.

**Figure 2:**
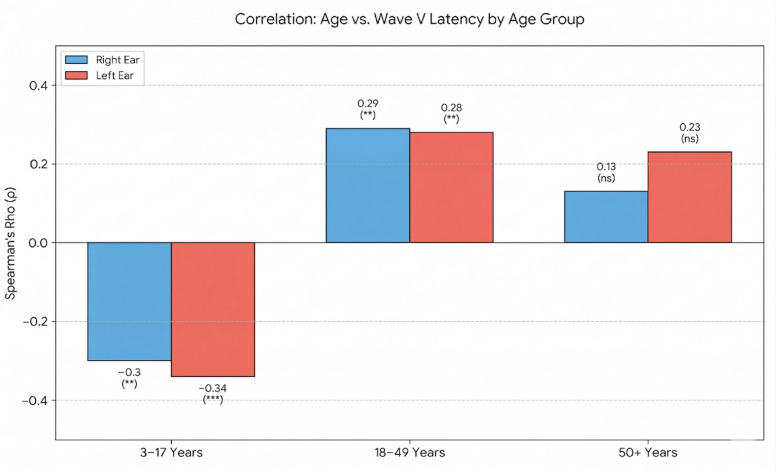
The correlation of age versus wave V latency by age group. ** *p* < 0.01; *** *p* < 0.001; ns = not statistically significant (*p* ≥ 0.05).

In the oldest group (50+ years), the correlations were positive but weak and did not reach statistical significance (right: *rho* = 0.13; left: *rho* = 0.23), indicating no reliable age-related trend in wave V latency within this subgroup (Figure 2). Overall, the pattern reflects a developmental shortening of wave V latency in childhood and adolescence, followed by a gradual lengthening in adulthood.

### 3.4. Effect of Stimulus Ear

**Figure 3:**
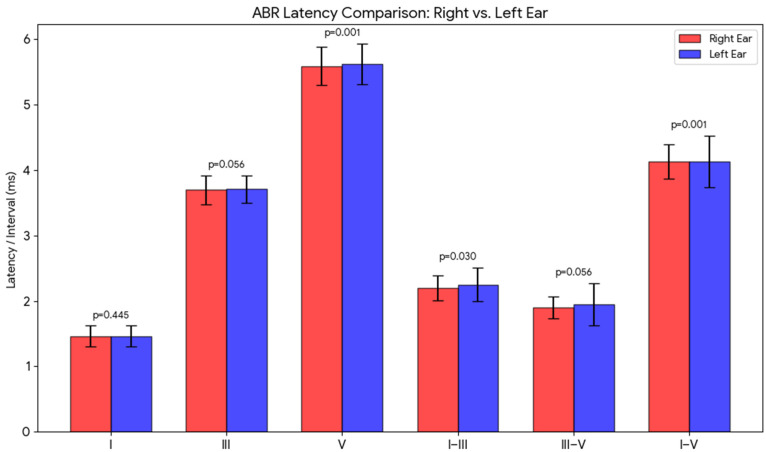
ABR latency comparison of right versus left ear.

As shown in Table 4 and Figure 3, there were no consistent right versus left ear differences for the absolute latencies of ABR waves, or for inter-wave latency intervals. According to the statistical analysis, there are significant differences for Wave V, inter-wave intervals I–III, and I–V. These differences may have been discovered as a result of the large-scale sample (*n* = 244 per ear) that was used in the current study. However, these differences might have been overlooked in samples that contained a smaller number of individuals.

Amplitude data for ABR stimulation of the right and left ears is shown in Table 5. There were no ear differences in the amplitudes of waves I and V or in the ratio of their amplitudes.

### 3.5. Expected Normative Values

Table 6 lists expected normative values in ms of latencies (lower limit and upper limit) for ear, gender, and age group based on median and interquartile range. The list includes absolute latencies of waves I, III, and V as well as interpeak intervals I–III and III–V, all at 80 dB nHL.

Table 7 lists expected normative values based on 2 standard deviations in the distributions of our findings. It shows absolute latencies of waves I, III, and V, values of interpeak intervals I–III, III–V, and I–V, interaural difference in wave V latencies, amplitudes of waves I and V, and the amplitude ratio of V/I, all at 80 dB nHL.

## 4. Discussion

The results of the present large-scale study replicate and expand upon the previous findings of Sanfins et al. (2022) [3] regarding significant sex-related differences in the assessment of click ABRs using the Neurosoft equipment (Ivanovo, Russia) version 1.0.107.1.

### 4.1. Effect of Sex

A statistically significant difference in latencies (waves III and V) and interpeak intervals (I–III, III–V, and I–V) was identified between the sexes, with women showing lower values than men. These findings are consistent with studies by Sanfins et al. (2022) [3], Krizman et al. (2012) [16], Lofti and Abdollahi (2012) [17], and Jerger and Hall (1980) [10] which all indicate a pattern of reduced latencies in women compared to men. Sexual differences have been observed throughout the auditory system’s trajectory, especially for the encoding of short-duration clicks [3,10,18] as well as other types of auditory stimuli [19]. One explanation for the presence of these differences is differing head size, with women having a smaller cranial vault than men [20]. However, the difference between men and women persists even with similar head sizes [21], reinforcing the idea that auditory information propagates faster in women [22].

Another notable aspect is that women exhibited greater standard deviations in the latencies of waves III and V compared to men, indicating a greater dispersion in female responses. These findings may be related to female hormones, as hormonal changes during menstrual cycles can modify auditory responses and, hence, perhaps click-ABRs [9,16]. The variability suggests that additional ABR studies should be conducted on women, taking into account different phases of the menstrual cycle, the use of hormonal contraceptives, and perhaps pregnancy, breastfeeding, perimenopause, and menopause. The current study did not control for these variables, which may have been the cause of greater variability among the group of women.

For wave I, the mean values and the standard deviations of males and females were similar, indicating that differences between the sexes emerge only at later stages. The absence of statistically significant differences in wave I latency between the sexes is a recurring finding in the literature [23,24]. In a study by Boer et al. (2022) [25], the latencies of wave I in both men and women were almost identical, similar to the findings of the present study. This finding may relate to the origin of this wave, the distal part of the auditory nerve. Since this region is more peripheral, differences in neural conduction speed between the sexes would be very small. However, further along the pathway, the differences in latency of subsequent waves would reflect differences in neuronal conduction delays resulting from differences in brain volumes.

### 4.2. Effect of Age

One aspect of the present study is the differences in responses between age groups, particularly prolongation of the latency of wave I in both ears. Wave I is generated by the synchronization of the distal portion of the auditory nerve, and these findings suggest a slowdown in neural conduction, a factor discussed in other studies [26,27]. According to Torre and Giraldo [26], there is a tendency for wave I to increase with age, in contrast with the latencies of waves III and V. The findings of the present study suggest that monitoring the latency of wave I might serve as a sensitive marker for the progressive aging of the auditory periphery, probably due to the loss of nerve fibers and a reduction in myelination.

We found that the latency of wave III remained relatively stable with age, which corroborates other studies which have identified the same maturational effect [28,29]. The stable responses of wave III across age demonstrate that the cochlear nuclei mature at around 24–36 months, and remain stable until old age [27]. This constancy of response can aid in the early diagnosis of different pathologies, as the presence of wave III within the normative values established for each equipment usually indicates functional integrity [30,31].

The maturational effect for wave V has already been reported by other studies, which confirm the presence of a shortening of wave V in the left ear, especially in childhood and adolescence (3–17 years). Apparently, myelination and synaptic activation bring about a more intense neural synchronization in this particular age group [30]. However, an inverse effect occurs among young adults (18–49 years), with the prolongation of wave V latencies, possibly due to the loss of oligodendrocytes or oxidative stress [32]. In the elderly (>50 years), however, there is only a weak positive trend.

The biphasic pattern in the latencies of wave V indicates a negative correlation during childhood and adolescence, reflecting a progressive shortening of this component, while there was a positive correlation in adulthood due to a progressive prolongation, and no significant correlation in old age. This provides valuable insights into neural maturation and senescence through a readily accessible and cost-effective assessment tool. Consequently, the assessment of click ABRs may facilitate the observation of both subclinical and clinical neurodegenerative phenomena.

### 4.3. Effect of Stimulus Ear

Differences in responses between the right and left ears were identified in the latencies of wave V, as well as in the interpeak intervals I–III and I–V. The analysis revealed a consistent pattern of interaural asymmetry, with the left ear showing prolonged responses compared to the right. These findings are consistent with the literature showing that a click-type stimulus is processed quicker in the right ear than in the left [33,34]. According to Sininger and Cone-Wesson (2006) [35], the lateral asymmetry in click-ABR originates from higher areas of the brainstem, corroborating the present study which observed changes in the latencies of wave V between left and right ears.

Auditory asymmetry has been studied in neuroimaging studies, where the dominance of the right ear over the left has been confirmed; this factor could explain the differences in the values of interpeak intervals I–III and I–V [36]. In such a case, individuals with hearing thresholds within the normal range show a preference for the right ear in the analysis of sound stimuli, which leads to better performance of this ear [37,38]. Thus, sound perception begins to be activated in regions of the brainstem that are involved in generating click-ABRs. The structures responsible for more peripheral functions transmit information to higher areas of the auditory system, the corpus callosum and more central areas [39].

We found there were no differences between the ears in terms of the amplitudes of waves I and V, the amplitude ratio of V/I, and the interaural differences in wave V. This symmetry reflects the usual neural synchrony and highlights that any asymmetry might indicate the presence of unilateral pathology [40,41].

### 4.4. The Influence of Sex, Ear, and Age on the ABR

The differences in responses between sexes, ears, and age in the present large-scale study reinforce the importance of using normative values for click-ABR. In such work, one important variable is the equipment used, in this case the NeuroAudio device manufactured by Neurosoft (Ivanovo, Russia). Different equipment will give different results and potentially compromise neurodiagnostic investigations. The results of the present study support that ABR analysis should be stratified by age, as this enables maturational and degenerative processes to be differentiated. Nevertheless, longitudinal studies on a larger scale are needed to refine our findings in the elderly, where subject numbers were low. The reduced sample size in this age group may not fully capture the natural variability of ABR parameters associated with presbycusis and other age-related neurological changes. Therefore, future large-scale longitudinal studies with targeted recruitment of elderly individuals are warranted to consolidate and refine the normative database for click-ABR

## 5. Conclusions

This study validates the significant effect of sex on the temporal parameters of the ABR and also provides new evidence supporting the idea that this effect does not extend to wave amplitudes. The identification of significant interaural asymmetries introduces another critical variable for clinical consideration. These findings reinforce the need to use sex-specific data, as well as ear-specific and age-normative data, to maximize the diagnostic accuracy of click-ABRs recorded using the NeuroAudio equipment. Nevertheless, it would be interesting for the study to have continuity in order to verify that the differences that were discovered are maintained even with the increase in the number of individuals.

## Figures and Tables

**Table 1:** Differences between men and women in terms of absolute wave latencies and inter-wave latency intervals.

	Men (*n* = 134)		Women (*n* = 110)		*U*	*p*
	*M*	*SD*	*M*	*SD*		
RE I	1.47	0.15	1.46	0.16	6999.0	0.499
RE III	3.73	0.18	3.65	0.25	**5297.5**	**<0.001**
RE V	5.65	0.25	5.55	0.32	**4992.5**	**<0.001**
RE I–III	2.26	0.16	2.19	0.23	**5109.0**	**<0.001**
RE III–V	1.93	0.16	1.86	0.16	**5609.5**	**0.001**
RE I–V	4.19	0.22	4.05	0.28	**4561.0**	**<0.001**
LE I	1.46	0.15	1.46	0.17	7027.5	0.930
LE III	3.76	0.19	3.64	0.21	**4691.0**	**<0.001**
LE V	5.71	0.29	5.52	0.31	**4315.0**	**<0.001**
LE I–III	2.32	0.31	2.18	0.18	**4250.0**	**<0.001**
LE III–V	2.07	0.38	1.88	0.20	**5388.0**	**0.002**
LEI I–V	4.20	0.45	4.05	0.29	**4128.5**	**<0.001**

Key: RE, right ear; LE, left ear; *M*, mean; *SD*, standard deviation; *U*, result of Mann–Whitney test; *p*, actual *p*-value. Bold indicates statistical significance (*p* ≤ 0.05).

**Table 2:** Correlations (rho Spearman) between age (years) and wave V latency.

Age Group	RE_V	LE_V
3–17 years	−0.30 **	−0.34 ***
18–49 years	0.29 **	0.28 **
50+ years	0.13	0.23

Key: ** *p* < 0.01; *** *p* < 0.001.

**Table 3:** Correlations (rho Spearman) between age (years) and absolute latency values of waves I, III, and V and interpeak intervals I–III, III–V, and I–V for both ears.

	Age
RE_I	0.21 **
RE_III	−0.09
RE_V	−0.09
RE_I_III	−0.31 ***
RE_III_V	−0.05
RE_I_V	−0.23 ***
LE_I	0.33 ***
LE_III	−0.03
LE_V	−0.14 *
LE_I_III	−0.30 ***
LE_III_V	−0.19 **
LE_I_V	−0.32 ***

* *p* < 0.05; ** *p* < 0.01; *** *p* < 0.001.

**Table 4:** Differences (ms) for absolute latencies and inter-wave latency intervals between the right ear and the left ear.

	Right Ear (*n* = 244)		Left Ear (*n* = 244)		*W*	*p*
	*M*	*SD*	*M*	*SD*		
I	1.46	0.16	1.46	0.16	0.77	0.445
III	3.70	0.22	3.71	0.21	1.91	0.056
V	5.59	0.29	5.62	0.31	**3.30**	**0.001**
I–III	2.20	0.19	2.25	0.26	**2.17**	**0.030**
III–V	1.90	0.17	1.95	0.32	1.91	0.056
I–V	4.13	0.26	4.13	0.39	3.24	0.001

Key: *M*, mean; *SD*, standard deviation; *W*, result of Wilcoxon test; *p*, actual *p*-value. Bold indicates statistical significance (*p* ≤ 0.05).

**Table 5:** Differences in the amplitudes for waves I and V (µV), ratio of the amplitude V/I, and interaural difference (IAD) of the wave V amplitudes for ABRs elicited by stimulation of the right or left ear.

	Right Ear (*n* = 244)		Left Ear (*n* = 244)		*W*	*p*
	*M*	*SD*	*M*	*SD*		
I	0.36	0.18	0.37	0.19	0.82	0.412
V	0.50	0.20	0.51	0.24	0.72	0.472
V/I	1.66	0.89	1.64	1.10	0.62	0.538
IAD V	0.08	0.07	0.08	0.07	0.00	>0.999

Key: *M*, mean; *SD*, standard deviation; *W*, result of Wilcoxon test; *p*, actual *p*-value.

**Table 6:** Expected normative values in ms of latencies (lower limit and upper limit) for ear, gender, and age group based on median and interquartile range. The list includes absolute latencies of waves I, III, and V as well as interpeak intervals I–III and III–V, all at 80 dB nHL.

Ear	Gender	Age Group	I	III	V	I–III	III–V	I–V
Right	M	3–17	1.24–1.62	3.52–3.95	5.17–5.99	2.08–2.49	1.66–2.16	3.76–4.54
		18–49	1.28–1.79	3.49–3.98	5.38–5.90	2.01–2.41	1.69–2.13	4.03–4.28
		50+	1.36–1.88	3.45–4.09	5.38–6.04	1.93–2.50	1.78–2.06	3.82–4.40
	F	3–17	1.28–1.59	3.38–4.03	5.06–5.97	1.98–2.46	1.65–2.10	3.61–4.43
		18–49	1.24–1.62	3.32–3.82	5.01–5.80	1.94–2.34	1.62–2.05	3.69–4.26
		50+	1.24–1.65	3.50–3.96	5.32–5.94	1.83–2.52	1.75–2.10	3.93–4.20
Left	M	3–17	1.27–1.59	3.51–4.02	5.22–6.06	2.06–2.58	1.69–2.18	3.81–4.62
		18–49	1.34–1.68	3.53–4.03	5.38–5.90	2.04–2.47	1.72–2.07	3.88–4.47
		50+	1.28–1.85	3.51–4.22	5.45–6.19	2.09–2.42	1.69–2.15	4.00–4.36
	F	3–17	1.24–1.66	3.41–3.88	5.20–5.92	1.94–2.41	1.71–2.11	3.75–4.47
		18–49	1.20–1.76	3.25–3.89	5.03–5.77	1.97–2.35	1.63–2.11	3.61–4.25
		50+	1.34–1.84	3.50–4.02	5.38–5.90	1.84–2.50	1.64–2.12	3.94–4.30

Key: M—male; F—female; ms—milliseconds.

**Table 7:** Expected normative values in ms of latencies (lower limit and upper limit) for ear, gender, and age group based on 2 standard deviations. The list includes absolute latencies of waves I, III, and V as well as interpeak intervals I–III and III–V, all at 80 dB nHL.

Ear	Gender	Age Group	I	III	V	I–III	III–V	I–V
Right	M	3–17	1.17–1.71	3.37–4.08	5.10–6.20	1.97–2.61	1.61–2.25	3.74–4.69
		18–49	1.24–1.80	3.40–4.05	5.20–6.03	1.93–2.45	1.56–2.33	3.74–4.49
		50+	1.25–1.90	3.44–4.16	5.31–6.16	1.91–2.54	1.69–2.17	3.79–4.53
	F	3–17	1.15–1.74	3.05–4.39	4.79–6.42	1.66–2.89	1.50–2.27	3.40–4.91
		18–49	1.11–1.80	3.25–3.94	4.97–5.89	1.86–2.42	1.55–2.11	3.61–4.33
		50+	1.17–1.87	3.31–4.17	5.23–6.08	1.72–2.71	1.67–2.16	3.75–4.51
Left	M	3–17	1.15–1.70	3.35–4.13	5.07–6.37	1.64–3.06	1.16–2.96	3.14–5.31
		18–49	1.22–1.80	3.38–4.14	5.23–6.05	1.89–2.61	1.63–2.11	3.73–4.52
		50+	1.27–1.91	3.44–4.22	5.35–6.24	1.97–2.53	1.59–2.33	3.88–4.54
	F	3–17	1.14–1.74	3.21–4.10	4.89–6.32	1.88–2.56	1.46–2.44	3.44–4.88
		18–49	1.13–1.80	3.20–4.01	4.95–5.92	1.79–2.49	1.53–2.12	3.53–4.41
		50+	1.09–2.06	3.41–4.18	5.20–6.22	1.81–2.63	1.56–2.27	3.86–4.41

Key: M—male; F—female; ms—milliseconds.

## Data Availability

The raw data supporting the conclusions of this article will be made available by the authors upon request due to privacy concerns.

## Abbreviations

The following abbreviations are used in this manuscript:

ABR	Auditory Brainstem Response
daPa	Decapascal
dB	decibels
dBHL	Decibel Hearing Level
dBnHL	Decibel Normalized Hearing Level
EPM	Paulista School of Medicine
ER–3A	Etymotic Research Insert Earphones Model 3A
Fz	Frontal Zero Electrode Position
FFR	Frequency Following Response
GSI	Grason-Stadler Inc.
Hz	Hertz
IAD	Interaural Asymetry Difference
ICF	Informed Consent Form
IEC-645	International Electrotechnical Commission Standard 645
ISO-389	International Organization for Standardization 389
kHz	Kilohertz
kΩ	Kilo-ohm
LE	Left Ear
M	mean
MN	Minnesota
Mmhos	Millimhos
Ms	millisecond
RE	Right Ear
SD	Standard Deviation
SRT	Speech Recognition Threshold
SPSS	Statistical Package for the Social Sciences
TM	Trademark
UNIFESP	Federal University of São Paulo
USA	United States of America
WHO	World Health Organization
µV	Microvolt
